# Dissecting asthma pathogenesis through study of patterns of cellular traffic indicative of molecular switches operative in inflammation

**DOI:** 10.7603/s40855-015-0001-2

**Published:** 2015-12-25

**Authors:** Ena Ray Banerjee

**Affiliations:** Department of Zoology, University of Calcutta, 35, Ballygunge Circular Road, 700019 Kolkata, West Bengal India

**Keywords:** **Bone marrow**, **Mesenchymal stem cell**, **GMP**, **Serum free medium**, **Platelet rich plasma**

## Abstract

**Background:**

Inflammation and degeneration are the two edged swords that impale a pulmonary system with the maladies like asthma and idiopathic pulmonary fibrosis. To explore critical role players that orchestrate the etiology and pathogenesis of these diseases, we used various lung disease models in mice in specific genetic knockout templates.

**Materials and methods:**

Acute and chronic allergic asthma and idiopathic pulmonary fibrosis model in mouse was developed in various genetic knockout templates namely α4^Δ/ Δ^(α41-/-), β2-/-, and α4-/- β2 mice, and the following parameters were measured to assess development of composite asthma phenotype- (i) airway hyperresponsiveness to methacholine by measuring lung resistance and compliance by invasive and P_enh_ by non-invasive plethysmography as well as lung resistance and compliance using invasive plethysmography, (ii) *in situ* inflammation status in lung parenchyma and lung interstitium and also resultant airway remodelling measured by histochemical staining namely Masson’s Trichrome staining and Hematoxylin&Eosin staining, (iii) formation of metaplastic goblet cells around lung airways by Alcian blue dye, (iv) measurement of Th1 and Th2 cytokines in serum and bronchoalveolar lavage fluid (BALf), (v) serum allergen-specific IgE. Specifically, ovalbumin-induced acute allergic asthma model in mice was generated in WT (wildtype) and KO (knockout) models and readouts of the composite asthma phenotype viz. airway hypersensitivity, serum OVA-specific IgE and IgG, Th2 cytokine in bronchoalveolar lavage fluid (BALf) and lymphocyte cell subsets viz. T, B cells, monocytes, macrophages, basophils, mast cells and eosinophils (by FACS and morphometry in H&E stained cell smears) were assessed in addition to lung and lymph node histology.

**Results:**

We noticed a pattern of cellular traffic between bone marrow (BM)→ peripheral blood (PB) → lung parenchyma (LP) → (BALf) in terms of cellular recruitment of key cell sub-types critical for onset and development of the diseases which is different for maintenance and exacerbations in chronic cyclically occurring asthma that leads to airway remodelling. While inflammation is the central theme of this particular disease, degeneration and shift in cellular profile, subtly modifying the clinical nature of the disease were also noted. In addition we recorded the pattern of cell movement between the secondary lymphoid organs namely, the cervical, axillary, ingunal, and mesenteric lymph nodes vis-à-vis spleen and their sites of poiesis BM, PB and lung tissue. While mechanistic role is the chief domain of the integrins (α4 i.e. VLA-4 or α4β1, VCAM-1; β2 i.e. CD18 or ICAM-1).

**Concluding remarks:**

The present paper thoroughly compares and formulates the pattern of cellular traffic among the three nodes of information throughput in allergic asthma immunobiology, namely, primary lymphoid organs (PLO), secondary lymphoid organs (SLO), and tissue spaces and cells where inflammation and degeneration is occurring within the purview of the disease pathophysiological onset and ancillary signals in the above models and reports some interesting findings with respect to adult lung stem cell niches and its resident progenitors and their role in pathogenesis and disease amelioration.

## Introduction

Inflammation is meant to re-establish a shift in the body’s homeostatic balance. Acute inflammation is the initial response to harmful stimuli and is achieved by the increased movement of plasma and leukocytes from blood into injured tissues concomitant with a cascade of biochemical events involving the systemic role of vascular and immune system and local role of other tissue specific cells within the injured tissue (ZF, 2007). Prolonged inflammation or chronic inflammation, leads to a progressive shift in the type of cells present at the site of inflammation and is characterized by simultaneous destruction and healing of the tissue from the inflammatory process. This is characterized by concurrent active inflammation, tissue destruction, and attempts at repair and may not be typically characterized by the aforementioned classic signs of acute inflammation. Instead, chronically inflamed tissue is characterized by the infiltration of mononuclear immune cells (monocytes, macrophages, lymphocytes, and plasma cells), tissue destruction, and attempts at healing, which include angiogenesis and fibrosis (Yang, 2006).

Asthma and COPD although differ significantly in their underlying etiology, involve similar inflammatory changes in the respiratory tract. While the specific nature and the reversibility of these processes largely differ in each entity and disease stage, both are characterized by lung inflammation; though patients with asthma suffer largely from reversible airflow obstruction, whereas patients with COPD experience a continuous decline in lung function as disease progresses (Davidson et al., 2010). By 2020 India alone will account for 18% of the 8.4 million tobacco related deaths globally (Sharma P, 2009). In China, COPD is one of the high frequency causes of death followed closely by Ischemic heart disease and cardiovascular disease (Broide et al., 1998).

Inflammation is therefore key to etiology of most respiratory disorders and while it is critical for the body’s defence against infections and tissue damage, it has increasingly become clear that there is a fine balance between the beneficial effects of inflammation cascades and potential for tissue destruction in the long term. If they are not controlled or resolved, inflammation cascades lead to development of diseases such as chronic asthma, rheumatoid arthritis, psoriasis, multiple sclerosis and inflammatory bowel disease (Takizawa, 2007).

The specific characteristics of inflammatory response in each disease and site of inflammation may differ but recruitment and activation of inflammatory cells and changes in structural cells remain a universal feature. This is associated with increase in the expression of components of inflammatory cascade viz. cytokines, chemokines, growth factors, enzymes, receptors, adhesion molecules and other biochemical mediators.

The pathogenesis of allergic asthma involves the recruitment and activation of many inflammatory and structural cells, all of which release mediators that result in typical pathological changes of asthma. The chronic airway inflammation of asthma is unique in that the airway wall is infiltrated by T lymphocytes of the T-helper (Th) type 2 phenotype, eosinophils, macrophages/monocytes and mast cells. Accumulation of inflammatory cells in the lung and airways, epithelial desquamation, goblet cell hyperplasia, mucus hyper-secretion and thickening of submucosa resulting in bronchoconstriction and airway hyperresponsiveness are important features of asthma (Czarnobilska E, 2005; Murphy, 2010). Both cells from among the circulating leukocytes such as Th2 lymphocytes, mature plasma cells expressing IgE, eosinophils (Murphy, 2010) and neutrophils as well as local resident and structural cells constituting the ‘respiratory membrane’ (airway epithelial cells, fibroblasts, resident macrophages, bronchial smooth muscle cells, mast cells etc.) contribute to the pathogenesis of asthma (Henderson et al., 2002). Airway hyperresponsiveness of asthma is clinically associated with recurrent episodes of wheezing, breathlessness, chest tightness and coughing, particularly at night or in early morning. Furthermore, during exacerbations the features of “acute on chronic” inflammation have been observed. Chronic inflammation may also lead to the outlined structural changes often referred to as airway remodelling which often accounts for the irreversible component of airway obstruction observed in some patients with moderate to severe asthma and the declining lung function.

Inflammation in COPD is associated with an inflammatory infiltrate composed of eosinophils, macrophages, neutrophils, and CD8^+^ T lymphocytes in all lung compartments (Henderson et al., 2002) along with inflammatory mediators such as TNF-α, IL-8 (interleukin- 8), LTB4 (Leucotriene B4), ET-1 (Endothelin-1) and increased expression of several adhesion molecules such as ICAM-1 (Woodside and Vanderslice, 2008). The molecular mechanisms whereby inflammatory mediators are upregulated at exacerbation may be through activation of transcription factors such as nuclear factor (NF)- κB and activator protein-1 that increase transcription of proinflammatory genes (Erlandsen et al., 1993). Acute exacerbations have a direct effect on disease progression by accelerating loss of lung functional though the inflammatory response at exacerbation is variable and may depend in part on the etiologic agent (Poole, 2001; Troosters et al., 2010). Current therapies for COPD exacerbations are of limited effectiveness (Rennard et al., 2007).

Rational treatment depends on understanding the underlying disease process and there have been recent advances in understanding the cellular and molecular mechanisms that may be involved (Rennard et al., 2007). Beyond the absence of curative therapy, current treatment options have inherent limitations, such as further complication by exacerbations, limitations of some orally available treatments and even refractoriness of the most effective treatment regimens such as inhaled corticosteroids (ICSs), long-acting beta2-agonists (LABAs), methylxanthines, leukotriene modifiers, chromones, and IgE blockers. Oropharyngeal adverse events and inadequate response to ICS in a lot of patients present a threat to continued therapy (Calverley et al., 2007). Targeting oxidative damage using antioxidants such as N-acetylcysteine as shown efficacy in chronic bronchitis (Erlandsen et al., 1993) but is relatively ineffective in established COPD (Holgate, 2007). Targeting TNF-α to ameliorate inflammation has also been disappointing (Cushley et al., 1983; Nakajima, 1994). The use of inhaled steroids combined with longacting β2 agonists to reduce exacerbation rates in more severe disease is now widely accepted, but their effects on mortality are still in doubt (Laberge et al., 1995) and presently there are no effective strategies beyond smoking cessation to slow disease progression in horizon (Schneider et al., 1999). These data suggest that even relatively modest immunomodulators such as inhaled corticosteroids might further impact on local immunity already damaged by chronic inflammation and remodelling, rendering individuals to some degree more vulnerable to significant infections (Chin, 1997). Key to effective COPD therapy is prevention of loss of alveolar smooth muscle elasticity which is irreversible by early diagnosis and more effective intervention which is currently virtually non-existent.

It is with an objective to identify targets that conventional therapy has obviously overlooked or underrated, that the pattern of cellular traffic is being studied under various patho-physiological situations. A number of genetic knockout models of mice were used and tissue specific (lung) inflammation under asthmatic (Th1-driven) condition was explored and immune cell traffic from their site of poiesis to their site of pathophysiological manifestation were studied (Henderson et al., 1997).

In our work with various genotype knockout models of mice, the data generated and interpreted from detailed analyses of cells traveling between bone marrow, peripheral blood, lung parenchyma, airways and the different secondary lymph organs or lymphoid tissues (cervical, axillary, inguinal, mesenteric lymph nodes and Peyer’s patch), we have detected some specific patterns. This is a report on the pattern of cellular traffic from which certain cell subsets were discernible to play rate limiting roles which have been presented along with comments on molecular implications of such directed movement of these key cell types. When specific molecules, critical for signaling the onset and/or development and maintenance of patho-physiology of acute allergic asthma and the associated inflammatory changes, are absent, as in the genotype knockout models, cell trafficking is drastically altered. As apparent from the data presented in this paper, the pattern of cell traffic can actually be molecular signatures for diagnosis of molecular causes of etiology in particular pathological manifestations of acute allergic asthma.

The clear rationale for doing the work, that is meta-analysis of data and interpretation of cellular traffic are to understand-

1. when and which cells mobilize from the site of poiesis (primary lymphoid organs);
2. which cells are rate limiting for the sequence of steps required for onset, development, maintenance and exacerbation of acute asthma;
3. which residual cells come back from the focal region of inflammation to secondary lymphoid organs/tissues which may be critical for generating “central memory” cell pool;
4. key diagnostic as well as therapeutic differentiators in the etiology of several respiratory diseases with similar clinical symptoms.

Information obtained shall be key to devising therapeutic/prophylactic strategies by targeting small molecules (pharmacological intervention), cells (cell based therapy through tissue engineering), antibody induced neutralization or arrest of cell activation of specific cells (cell targeting) etc. for personalized and translational medical treatment. Unless specific targets are identified in a strict spatio-temporal format, interfering of a target leads to undesirable side effects or even fatality. As outlined in the initial paragraphs, there are patient populations which are refractory to some drugs. So even for designing combination therapy, the timing and targeting is important. The same cell may behave quite differently at different times of the disease onset. The same cell may express different cellular proteins or secrete soluble proteins in its milieu and such changes, rapid and occurring in sequence, are extremely critical information to catch the correct target and at the correct time. Work embodied in this paper attempts to elucidate just these nodes of information throughout in a cellular factory in a specific disease template, acute asthma.

## Materials-Methods

### Animals

C57BL6 mice were used as described previously (Banerjee, 2014; Banerjee and Henderson, 2012a; Banerjee and Henderson, 2012b; Banerjee and Henderson, 2013; Banerjee et al., 2009; Banerjee et al., 2012; Banerjee et al., 2008; Ray Banerjee, 2011a; Ray Banerjee, 2011b; Ulyanova et al., 2007). Mx.cre+α4 flox/flox mice were conditionally ablated by i.p. poly(I)poly(C) injection. *cre-* mice were used as WT (wild type) and α4 ablated mice were simply called α4-/-. CD18-/- mice on a C57BL6 background were called β2-/-. In total the following number of animals were used in each group: WT= 5 per experiment, +OVA= 5 per experiment, αa-/-= 5 per experiment, β2-/-= 5 per experiment, Rag2γC-/- (baseline)= 4 per experiment, Rag2γC-/- engrafted with WT BMC= 10 per experiment, Rag2γC-/- engrafted with α4-/- BMC= 10 per experiment. A total of three independent experiments for development and analyses of the OVA model and a total of four independent experiments for the engraftment and repopulation experiments in Rag2γC-/- mouse were performed. Data presented are mean ±SEM for all experiments and only p value less than 0.01 have been considered.

### Experimental design for lymphopoiesis

**Figure 1.**
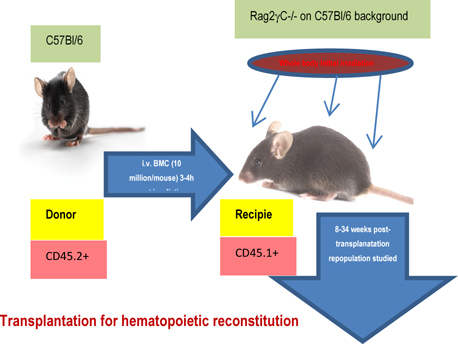
Study protocol for transplantation for hematopoietic reconstitution probing mobilization and homing.

5 million bone marrow cells in prewarmed HBSS were injected via tail vein in lethally irradiated (800 cGY) to 6-8 weeks old Rag2γC-/- recipients and reconstitution was followed at 5 weeks, 10 weeks and 6 months. Tissues were collected post sacrifice to assess the type of donorderived versus recipient’s own reconstituted cell types. In the repopulated animals, OVA-induced asthma was induced and composite asthma phenotype noted with detailed analysis of the cellular subtypes in the PLO, SLO and tissues- their structural identity and their functional propensity (**Fig**.**1**).

### Allergen sensitization and challenge

**Figure 2.**
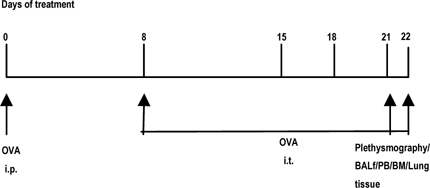
Study design to generate acute allergic asthma phenotype in mice.

Mice were sensitized and later challenged with OVA (Pierce, Rockford, IL) as described previously. Mice were immunized with OVA (100μg) complexed with aluminium sulfate in a 0.2-ml volume, administered by i.p. injection on day 0. On days 8 (250 μg of OVA) and on days 15, 18, and 21 (125μg of OVA), mice were anesthetized briefly with inhalation of isoflurane in a standard anesthesia chamber and given OVA by intratracheal (i.t.) administration. Intratracheal challenges were done as described previously. Mice were anesthetized and placed in a supine position on the board. The animal’s tongue was extended with lined forceps and 50 μl of OVA (in the required concentration) was placed at the back of its tongue. The control group received normal saline with aluminium sulfate by *i.p*. route on day 0 and 0.05 ml of 0.9% saline by i.t. route on days 8, 15, 18, and 21 (**Fig**. **2**).

### Pulmonary function testing

*In vivo* airway hyperresponsiveness to methacholine was measured 24 hours after the last OVA challenge in conscious, free moving, spontaneously breathing mice using whole-body plethysmography (model PLY 3211; Buxco Electronics, Sharon, CT) as previously described. Mice were challenged with aerosolized saline or increasing doses of methacholine (5, 20, and 40 mg/ml) generated by an ultrasonic nebulizer (DeVilbiss Health Care, Somerset, PA) for 2 min. The degree of bronchoconstriction was expressed as enhanced pause (P_enh_), a calculated dimensionless value, which correlates with the measurement of airway resistance, impedance, and intrapleural pressure in the same mouse. P_enh_ readings were taken and averaged for 4 min after each nebulization challenge. Penh was calculated as follows: P_enh_= [(Te/Tr-1)X (PEF/PIF), where T_e_ is expiration time, T_r_ is relaxation time, PEF is peak expiratory flow, and PIF is peak inspiratory flow X 0.67 coefficient. The time for the box pressure to change from a maximum to a user-defined percentage of the maximum represents the relaxation time. The T_r_ measurement begins at the maximum box pressure and ends at 40%.

#### BALf

After pulmonary function testing, the mouse underwent exsanguination by intra-orbital arterial bleeding and then BAL (0.4 ml three times) of both lungs.Total BAL fluid cells were counted from a 50μl aliquot and the remaining fluid was centrifuged at 200 *g* for 10 min at 4°C and the supernatants stored at –70°C for assay of BAL cytokines later. The cell pellets were re-suspended in FCS and smears were made on glass slides. The cells, after air drying, were stained with Wright-Giemsa (Biochemical Sciences Inc, Swedesboro, NJ) and their differential count was taken under a light microscope at 40X magnification. Cell number refers to that obtained from lavage of both lungs/mouse.

### Lung parenchyma

Lung mincing and digestion was performed after lavage as described previously with 100μ/ml collagenase for 1 hr at 37°C, and filtered through a 60# sieve (Sigma). All numbers mentioned in this paper refer to cells obtained from one lung/mouse.

### Lung histology

Lungs of other animals of same group were were fixed in 4% paraformaldehyde overnight at 4°C. The tissues were embedded in paraffin and cut into 5 μm sections. A minimum of 15 fields were examined by light microscopy. The intensity of cellular infiltration around pulmonary blood vessels was assessed by Hematoxylin and Eosin staining. Airway mucus was identified by staining with Alcian blue and Periodic Acid Schiff staining as described previously.

### Immunohistochemical staining of the lung

Lungs of yet other animals of the same group were processed for immunohistochemical staining following standard procedures. They were stained with either anti-VCAM-1 (MK2) or anti β1 (9EG7) antibody and colour development done by HRP. Mouse tissues were prefixed in 4% paraformaldehyde in 100 mM PBS (pH 7.4) for 6-12h at 4°C, washed with PBS 10 min 3 times and then the tissues were soaked in 10% sucrose in PBS for 2-3h, 15% sucrose in PBS for 2-3h, 20% for 3-12h at 4°C and then embedded in O.C.T. compound (Tissue-Tek 4583, Sakura Finetechnical CO., Ltd, Tokyo, 103, Japan) and frozen in acetone cooled by dry ice. Frozen blocks were stored at – 70°C refrigerator until sectioned. Frozen blocks were cut on a freezing, sliding macrotome at 4 μm (LEICA CM1850 Cryostat) and air-dried for 30 min at RT. After washing in PBS 3 times for 10 min at RT, to block endogenous peroxidase activity, 0.3% hydrogen peroxide was applied to each section for 30 min at RT. Each slide was incubated with blocking solution (normal serum from the specific secondary antibody was derived from) to block nonspecific reactions. Appropriately diluted primary antibody was applied to each slide and incubated for overnight at 4°C. After washing with PBS, slides were incubated with appropriately diluted specific biotin conjugated secondary antibody solution for 1h at RT. After washing with PBS, slides were incubated in AB reagent for 1h at RT (AB Complex/HRP, DAKO). After washing with PBS, slides were stained with 0.05% DAB (3,3’- diaminobenzidine tetrahydrochloride, Sigma) in 0.05 M Tris buffer (pH 7.6) containing 0.01% H_2_O_2_ for 5-40 min at RT.Slides were counterstained with Mayer’s hematoxylin and dehydrated in graded ethanol, xylene and mount with Mount-Quick (Daido Sangyo Co. Ltd., Japan).

### CFU-c assay

To quantitate committed progenitors, CFU-C assays were performed using methylcellulose semisolid media (Stemgenex, Amherst, NY) supplemented with an additional 50 ng of stem cell factor per ml (Peprotech, Rocky Hill, NJ) to promote growth of hematopoietic progenitors. Next, 0.01 x 10^6^ cells from lung were plated on duplicate 35-mm culture dishes and incubated at 37°C in a 5% CO_2_-95% air mixture in a humidified chamber for 7 days. Colonies generated by that time were counted using a dissecting microscope, and all colony types (i.e., BFU-E, CFU-E, CFU-G, CFU-GEMM, CFU-GM, and CFU-M) were pooled and reported as total CFU-C. Aliquots of 1-10 x 10^4^ cells were plated per 1 ml of semisolid methylcellulose (CFU-lite with Epo, Miltenyi Biotech, or complete human methylcellulose medium, Stem Cell Technologies, Vancouver, BC, Canada). CFU-C frequency was scored morphologically after 10 to 14 days in culture at 37°C, 5% CO_2_, in a humidified incubator.

### Fluorescein-activated cell sorter (FACS) analysis

Cells from hemolyzed peripheral blood (PB), bone marrow( BM), bronchoalveolar lavage (BAL), lung parenchyma (LP), spleen, mesenteric lymph nodes (MLN), cervical lymph nodes (CLN), axillary lymph nodes (LNX) and inguinal lymph nodes (LNI) were analyzed on a FACS Calibur (BD Immunocytometry Systems, San Jose, CA) by using the CELLQuest program. Staining was performed by using antibodies conjugated to fluorescein isothiocyanate (FITC), phycoerythrin (PE), allophycocyanin (APC), Peridinin Chlorophyll Protein (PerCP-Cy5.5) and Cy-chrome (PE-Cy5 and PE-Cy7). The following BD pharmingen (San Diego, CA) antibodies were used for cell surface staining: APC-conjugated CD45 (30F-11), FITC-conjugated CD3(145-2C11), PE-Cy5 conjugated CD4 (RM4-5), PE-conjugated CD45RC (DNL- 1.9), APC-conjugated CD8(53-6.7), PE-Cy5 conjugated B220 (RA3-6B2), FITC-conjugated IgM, PE-conjugated CD19 (ID3), PE-conjugated CD21(7G6), FITC-conjugated CD23 (B3B4), APC-conjugated GR-1(RB6-8C5), and PE-conjugated Mac1(M1/70). PE-Cy5 conjugated F4/80 (Cl:A3-1(F4/80)) was obtained from Serotec Ltd., Oxford, UK. PE-conjugated anti-α4 integrin (PS2) and anti-VCAM-1(M/K-2) was from Southern Biotechnology, Birmingham, Ala. Irrelevant isotype-matched antibodies were used as controls (Banerjee and Henderson, 2012b).

### ELISA for cytokines

Th2 cytokines (IL-4 and 5) and TNFα and IFNγ in BAL and serum (previously frozen at –70°C) were assayed with mouse Th1/Th2 cytokine CBA (BD Biosciences, San Diego, CA) following the manufacturer’s protocol. According to the manufacturer’s protocol, IL-13 and Eotaxin were measured by Quantikine M kits from R&D Systems, Minneapolis, MN (Banerjee, 2014).

### OVA specific IgE and IgG1 in ser

Anti-mouse IgE (R35-72) and IgG1 (A85-1) from BD Biosciences, San Diego, CA were used for measuring OVA specific IgE and IgG1 (in serum previously frozen at –70°C) respectively by standard ELISA procedures as previously described.

## Results

### Rationale for the study

The study was designed to develop a preclinical model of acute allergic asthma in C57Bl/6J mouse purchased from NIN under permission of the departmental animal ethics committee (approval dated 12-5-2010, renewed on 30-12-2013) and do a meta-analysis of unpublished earlier data generated in University of Washington, USA, (Banerjee, 2014; Banerjee and Henderson, 2012a; Banerjee and Henderson, 2012b; Banerjee and Henderson, 2013; Banerjee et al., 2009; Banerjee et al., 2012; Banerjee et al., 2008; Ray Banerjee, 2011a; Ray Banerjee, 2011b; Ray Banerjee, 2007; Ulyanova et al., 2007) together with new data generated in the University of Calcutta. Data from two main focused groups of experiments shall be shared and discussed in this work. Cell traffic from PLO to & from SLO to & from pulmonary tissue to & from PLO, post complete manifestation of composite asthma phenotype [some data published in which shall be further analyzed and discussed and new data presented here] and Lymphopoiesis, mobilization, homing and repopulation of PLO and SLO of lethally irradiated Rag2γC-/- recipients from WT (α4+/+) and αa ablated mouse bone marrow and then development (or not) of the composite asthma phenotype and inferences made thereof. [Here again some published data from shall be discussed in context with cellular traffic] α4 ablated and β2-/- mice were developed by other labs (Lee et al., 2003; Scott et al., 2003).

### α4+ cells in various tissues of α4^f/f^ and α4^Δ/Δ^ donors prior to transplantation

**Figure 3.**
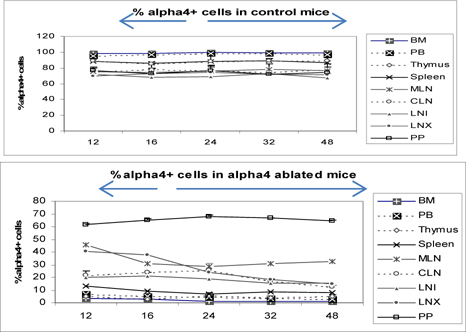
Distribution of alpha4+ cells in primary and secondary lymphoid organs: donor neonatally ablated Mx.cre alpha4-/- vs.

**Fig**. **3** shows the distribution of alpha4+ cells in alpha+ versus alpha4 ablated mice. This is in donor mice itself (the mice from which BMC was prepared from femur for engraftment into bloodstream. Understandably PB has the lowest number of alpha4+ cells while surprisingly Peyer’s patch has the highest number of alpha4+ cells in alpha 4 ablated mice. In WT BMC showed a greater homing and engraftment property than the KO BMC transplanted into the recipients. Cells that showed greatest (in terms of shortest time of migration and in terms of the greatest number of viable cells) homing and engraftment were the ones in PP, LNI and MLN. Of note, these are total number of cells including progenitors and differentiated functionally mature cells. These may be therefore labelled as the “leaky” tissues were postablation, α4 expression still occurs, designating the transcriptome in these tissues as being non-permissive to ablation.

### Clonogenic potential of bone marrow cells used in transplantation of Rag2γC-/- recipients

**Figure 4.**
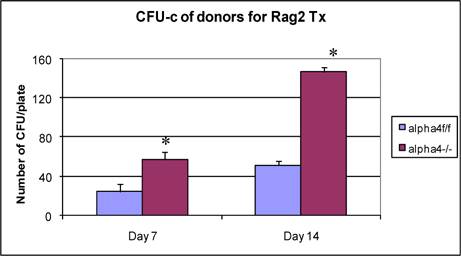
Donor BM CFU-C assessed before the transplant.

**Fig**. **4** presents clonogenic potential of bone marrow of WT vs. α4-/- mice. α4-/- bone marrow cells show greater colony forming potential as found earlier (Ray Banerjee, 2007; Scott et al., 2003; Ulyanova et al., 2007).

### Tissue distribution of hematopoietic progenitors posttransplantation and engraftment at 10 weeks

**Figure 5.**
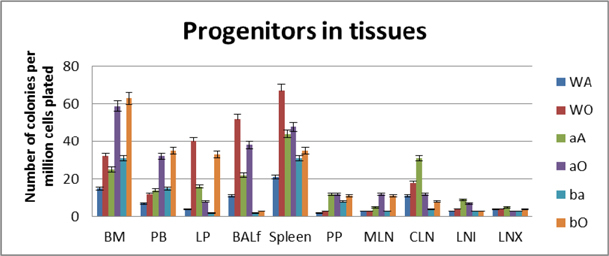
Progenitors in different tissues of the three genotype groups.

As seen from data presented in **Fig**. 5 PLO show the maximum variation among the genotype groups with or without OVA treatment in the PLO. Compared to WT, in both KO groups α4-/- and β2-/-, there was 2-fold, 2.3-fold and 2 fold increase in the number of progenitors in BM respectively post-OVA. In circulating blood, however, WT show 1.7-fold increase in circulating progenitor number compared to 2.3-fold in both KO groups. There was 3-fold increase in hematopoietic progenitors in spleen of WT compared to 9% and 13% respectively in α4-/- and β2-/- spleen. In the PP, WT and both KO groups show negligible change in progenitor number post-OVA. In tissue, a very curious thing is happening, compared to WT, where post-OVA increase in progenitor number in lung was 10-fold, in α4-/- it reduced half while in β2-/-, the number increased by 16.5-fold. In the interstitial spaces of the lung, from the BALf, the hematopoietic progenitors that were detected were 4.7-fold in WT compared to pre-OVA numbers being double in α4-/- but only a 1.7-fold increase post-OVA. BALf in β2-/- however were a negligible number and obviously demonstrates a mechanistic inability to migrate across the interstitium.

### Tissue distribution of mature and differentiated hematopoietic cells

Repopulation of thymus was significantly impaired in Rag 2–/– recipients of α4Δ/Δ cells, compared to those that received α4f/f donor cells (**Fig**. **6A**). A decrease in total cellularity (by 43% at 6 months) was again demonstrable at 8 months post-transplantation, indicating no restorative evidence with time posttransplantation. Double-positive (DP, CD4+/CD8+) population was the predominant one in Rag2–/– recipients of α4Δ/Δ or α4f/f donor cells. The CD4:CD8 ratio greatly favored the CD4 population (w8:1). This suggests that the total repopulation of thymus was impaired, likely because of impaired migration of BM-derived progenitors to thymus, although their subsequent maturation (to DP) was not grossly impaired in the absence of α4 integrins. However, it is notable that CD8+cells were at very low levels in thymus and lower than controls, in contrast to levels in PB (w1.9:1).

### Traffic between primary and secondary lymphoid tissue (differentiated immune cells only)

#### (a) Proximal circulation

Cellularity in cervical, axillary, and inguinal lymph nodes was similar to controls (i.e., recipients of α4f/f donor cells). Detailed evaluation of subset distribution showed that there were modestly decreased proportions of mature B cells (B220+IgM+) in all LNs tested, or decreased proportions of activated T cells (CD3+/CD25+, CD3+/CD44+), but their absolute numbers were not significantly different from control groups (**Fig**.**6** **B-I**). There was a tendency for preferential migration of CD45RC–/CD4+ (memory) cells to lymph nodes, whereas CD45RC+/CD4+ (naive) cells instead preferentially migrated to spleen and thymus in α4^Δ/Δ^ recipients. In spleen, as noted above, the cellularity, especially of red pulp, was significantly increased and concerned all developmental stages of B cells and of total T cells.

#### (b) Remote circulation

In Rag–/– recipients of α4^Δ/Δ^ cells both at 6 and 8 months post-transplantation, there was a significant reduction in cell numbers recovered from these tissues compared to controls (about 17-fold in PPs, 67% less in MLNs) (**Tables** **6B**-**I**). All subsets of B and T cells were severely reduced in PPs and MLNs repopulated by α4^Δ/Δ^ cells. The CD4:CD8 ratio in PPs favored a CD4+ profile, as seen in thymus. These data, like the ones in thymus, suggest significant homing impairment of all α4^Δ/Δ^ cells to these tissues.

### Rate limiting cells

From the above results and detailed cellular subpopulation analyses in **Tables** **1**-**16**, the following data stand out: In blood, WT lymphoid cells before OVA treatment increase to 1.75 folds after OVA treatment compared to non-lymphoid cells which shows insignificant increase. Whereas in α4-/-, they increase by 2.3-fold and in β2-/- increase is about 1.6-folds. In both KO mice, however, there is significant increase in number of nonlymphoid cells post-OVA compared to WT 5-fold in α4-/- and 1.6-fold in β2-/-. In lung, WT post-OVA lymphoid cells were a 106.6 fold compared to control but in only 26.57- fold more myeloid cells migrate to lung parenchyma due to OVA-induced inflammation. In both KO, non-lymphoid cells are 16-fold and 12-fold respectively compared to control. In BALf, there is 40-fold increase in number of lymphoid cells post-OVA in WT and 7.6-fold increase in myeloid cells, but other than these same cells showing a slight increase (3.5-fold) post-OVA in β2-/- cells, all KO cells basically failed to migrate across and show poor occurrence even compared to WT control (**Table** **1**). After the evaluation of the total and subpopulations of hematopoietic cells from PLO through blood to tissue site of inflammation (respiratory tissue), the obvious question was what fraction of the blood cells actually get recruited to SLO and thence to the inflamed tissue. **Tables** **3** onwards attempts to quantitate that in detail. Percent recruited from blood to tissue as shown in **Table** **2** show a post-OVA preferential recruitment of T cells in α4+ cells (60-fold) to α4- cells (28-fold) and none in β2- cells while B cells show a complete inability to migrate to tissues in both KO (decrease by 3-fold) compared to 2.5-fold increase in WT cells. Macrophages (GR1-F4/80+) show no significant change in β2-/- compared to WT and α4-/- recruitment (20-fold and 15-fold respectively). As for a population of cells expressing both the myeloid markers, although in WT the number of cells detected were small, there was significant increase in their recruitment from blood (133- fold) but in α4-/- mice, there was no difference in their recruitment post-OVA as opposed to β2-/- that showed feeble recruitment to the tune of thrice the number of cells recruited before OVA treatment which however was 166- fold higher to begin with in both KO groups.

As for the scenario in PLO (BM) other than T cells in whose number α4-/- cells show 10.6-fold increase in synthesis of T cells as opposed to B cells, myeloid cells and indeed all other CD45+ cells compared to WT where an average of 2.3±0.36 fold increase in seen post-OVA (**Table** **3**). **Tables** **4**-**7** **(A-D)** detail the count of different cell populations and is much more elaborate readout of **Table** **1**. The important highlight here is that lung parenchyma, BALf and trachea being the different structural components of the respiratory tissue, the last seems to be a key node of information throughput and compared to WT and β2-/- mice, α4-/- mice show a much exaggerated CD4:CD8 ratio 5:1 compared to 3:1 in the former. Additionally in all four tissues, **Tables** **5**-**7C**, all three species of B cells, viz. B220+IgM+ mature plasma cells, B220+CD19+ memory cells, B220+CD23+ allergen specific plasma cells are severely decreased in number in β2-/-mice *ab initio* and although there is significant increase in these numbers after OVA treatment, the overall cell number in this KO mouse in respiratory tissue falls far short of even the numbers of these cells in the placebo treated WT. **Table** **8B** shows that both KO mice show a Th2 skewed phenotype in untreated mice which respond to OVA in terms of ration but neither threshold number nor the composite phenotype is attained indicating that CD4:CD8 may not be absolutely essential for the etiopathophysiology at a critical level. The interesting finding in data presented in these levels however is probably the most important finding of the entire work because of the memory versus naïve or immature T and B cell numbers before and after OVA treatment (**Table** **8C**, **D**).

As regards the secondary lymphoid organs and tissues, the first to be considered was CLN as it is anatomically proximal to the lung. **Table 9A-C** displays an increase in total number of cells 26.5% and 31.3% were respectively contributed by T and B cells. Interestingly, post-OVA this increased by 5-folds in α4^Δ/Δ^ but remained same in β2-/-.

**Table 1.** Values represent total number of different cells and their subsets migrated from bone marrow (BM) via circulation that is peripheral blood (PB) to lung parenchyma (LP) and interstitium (bronchoalveolar lavage fluid- BALf). Recruitment of all leukocytes and their subsets is less in α4-/- lung as well as BALf compared to control. In β2-/- lung, except T cells and eosinophils, all other cells were increased in number. (*P<0.01 compared with post-OVA control) Recruited T cells post-OVA (both CD4+ and CD8+) were CD45RC negative (memory) in control lung and BAL but mostly CD45RC positive (na ïve) in α4- /- and β2-/- mice. Note the differences between α4 and β2 deficient mice in LP cell content. Mϕ denotes macrophage. (n=12/genotype group). (L=Lymphoid, NL+Non-lymphoid)

(x10^6^)			Blood	Lung	BAL	Trachea
**WT**	**Lymphoid**	**before**	8.01±1.3	0.015±0.001	0.06±0.01	0.004±0.001
		**after**	13.9±2.4	1.6±0.05	2.4±0.4	0.25±0.15
	**Non-Lymphoid**	**before**	3.7±0.7	0.07±0.001	0.8±0.003	0.013±0.01
		**after**	4.3±1.2	1.86±0.06	6.1±0.098	0.06±0.03
**α4-/-**	**Lymphoid**	**before**	19±3.2	0.015±0.001	0.02±0.009	0.012±0.005
		**after**	44±1.1	0.3±0.001	0.05±0.001	0.086±0.07
	**Non-Lymphoid**	**before**	6.8±2.1	0.05±0.001	0. 6+0.016	0. 6+0.016
		**after**	34.8±3.3	1.022±0.05	0.8±0.001	0.2±0.1
**β2-/-**	**Lymphoid**	**before**	35.9±5	0.006±0.0001	0.021±0.01	0.014±0.001
		**after**	57.9±8.2	0.104±0.05	0.08±0.03	0.08±0.02
	**Non-Lymphoid**	**before**	50.2±8.3	0.43±0.07	0.18±0.001	0.16±0.09
		**after**	79.6±5.1	5.18±0.03	0.64±0.03	0.46±0.2

**Table 2A.** Values represent percent cells of blood to be found in various tissues. The percentage blood cells that migrated to interstitial spaces of the lung (BALf). Number of cells in PLO and SLO and tissue were calculated as tC and DC by hemocytometric analysis in a standard Neubauer’s Hemocytometer, DC was analyzed by double blind counting from H&E stained smears prepared in a cytospin (manufactured by Vision Scientific, South Korea, model Centurion Scientific C2 series) using Zeiss photo allotment and Axiostar plus software and by flow cytometry using BD flow cytometer (BD Accuri C6 cytometer) and analyzed by BD Accuri C6 software using monoclonal fluorochrome tagged antibody as mentioned in Materials & Methods.

	WT		α4-/-		β2-/-	
	Before	After	Before	After	Before	After
**Total cells**	7.29	46.6	2.4	1.03	0.2	0.5
**T cells**	0.17	14	0.014	0.05	0.02	0.14
**B cells**	1.68	22	0.28	0.15	0.35	0.136
**GR1-F4/80+**	0.7	27.6	0.07	0.09	0.4	2.3
**GR1+F4/80+**	13.5	21.5	0.5	2.3	0.3	0.7
**GR1loF4/80hi**	7.6	16.5	0.5	2.3	2.6	7.7
**GR1loF4/80lo**	16	13.8	0.5	1.2	0.5	0.6
**GR1hiF4/80lo**	9.6	73	0.04	2.16	0.03	0.35
**GR1+F4/80-**	15	46	0.6	0.3	0.06	0.045

**Table 2B.** Values represent percent cells of blood to be found in various tissues. The percentage blood cells that migrated to lung parenchyma. Number of cells in PLO and SLO and tissue were calculated as tC and DC by hemocytometric analysis in a standard Neubauer’s Hemocytometer, DC was analyzed by double blind counting from H&E stained smears prepared in a cytospin (manufactured by Vision Scientific, South Korea, model Centurion Scientific C2 series) using Zeiss photo allotment and Axiostar plus software and by flow cytometry using BD flow cytometer (BD Accuri C6 cytometer) and analyzed by BD Accuri C6 software using monoclonal fluorochrome tagged antibody as mentioned in Materials & Methods.

	WT		α4-/-		β2-/-	
	Before	After	Before	After	Before	After
**Total cells**	0.75	19.7	0.27	1.67	0.55	3.87
**T cells**	0.26	18.3	0.09	1.1	3	0.009
**B cells**	0.14	0.54	0.07	0.05	0.16	0.7
**GR1-F4/80+**	0.05	1.05	0.017	0.11	0.42	1.875
**GR1+F4/80+**	0.88	31	0.48	1.76	0.8	5.6
**GR1loF4/80hi**	0.04	6.15	0.12	0.2	5.4	28
**GR1loF4/80lo**	1.9	18.5	1.25	0.89	1.26	6.4
**GR1hiF4/80lo**	7	1.466	6	0.9	0.3	5.7
**GR1+F4/80-**	0.3	39.5	0.1	1.1	0.1	1.67

**Table 2C.** Values represent percent cells of blood to be found in various tissues. The percentage of blood cells that migrated to trachea. Number of cells in PLO and SLO and tissue were calculated as tC and DC by hemocytometric analysis in a standard Neubauer’s Hemocytometer, DC was analyzed by double blind counting from H&E stained smears prepared in a cytospin (manufactured by Vision Scientific, South Korea, model Centurion Scientific C2 series) using Zeiss photo allotment and Axiostar plus software and by flow cytometry using BD flow cytometer (BD Accuri C6 cytometer) and analyzed by BD Accuri C6 software using monoclonal fluorochrome tagged antibody as mentioned in Materials & Methods.

	Before	After	Before	After	Before	After
**Total cells**	1.07	1.35	0.2	0.2	0.3	0.3
**T cells**	0.05	3	0.013	0.37	0.02	0.08
**B cells**	0.07	0.176	0.18	0.06	0.15	0.3
**GR1-F4/80+**	0.01	0.2	0.04	0.6	0.6	0.5
**GR1+F4/80+**	0.0003	0.04	0.7	0.3	0.5	1.5
**GR1+F4/80-**	0.001	0.17	0.3	0.4	0.08	0.2

**Table 3.** Lymphoid and myeloid cells in bone marrow. Number of cells in PLO and SLO and tissue were calculated as tC and DC by hemocytometric analysis in a standard Neubauer’s Hemocytometer, DC was analyzed by double blind counting from H&E stained smears prepared in a cytospin using Zeiss photo allotment and Axiostar plus software and by flow cytometry using BD flow cytometer and analyzed by BD Accuri C6 software using monoclonal flurochrome tagged antibody as mentioned in Materials & Methods.

Genotypes	Total (x10^6^)	CD45+(x10^6^)	T cells(x10^6^)	B cells(x10^6^)	Myeloid cells(x10^6^)
**WT+alum**	28.07±8.3	25.86±7.6	0.9±0.05	2.485±0.1	22.475±10.3
**WT+OVA**	54.13±11.6	46.5±2.6	1.895±0.75	6.49±1.6	38.085±9.2
**α4-/-+alum**	34.3±6.5	28.94±5.7	0.62±0.2	3.675±1.4	24.645±1.3
**α4-/-+OVA**	104.15±8.8	85.34±11.9	6.57±0.5	7.7±0.7	71.07±18.9
**β2-/-+alum**	82.05±11.5	67.7±1.05	2.7±0.9	9.7±0.54	55.3±1.15
**β2-/-+OVA**	108.17±13.2	96.7±13.3	4.15±1.3	10.4±1.6	82.15±4.6

**Table 4A.** Total count (Lymphoid and myeloid cells) in Blood. Number of cells in PLO and SLO and tissue were calculated as tC and DC by hemocytometric analysis in a standard Neubauer’s Hemocytometer, DC was analyzed by double blind counting from H&E stained smears prepared in a cytospin using Zeiss photo allotment and Axiostar plus software and by flow cytometry using BD flow cytometer and analyzed by BD Accuri C6 software using monoclonal flurochrome tagged antibody as mentioned in Materials & Methods.

(x10^6^)		Total	T cells	B cells
**WT**	**Control**	11.92±2.4	5.21±1.3	2.8±0.6
	**OVA treated**	18.22±6.4	7.69±2.4	6.25±1
**α4-/-**	**Control**	26.2±8.8	13.8±3.2	5.32±1.7
	**OVA treated**	79.04±22.2	18.5±1.1	25.6±7.5
**β2-/-**	**Control**	87.5±19.8	32.2±5	3.7±2.3
	**OVA treated**	138±60.1	43.3±8.2	14.6±1.5

**Table 4B.** Blood

T cell subset								
(x10^6^)		CD4+	CD8+	CD4+		CD8+		CD4:CD8
				memory	naive	memory	naive	
**WT**	**Control**	4.34±1.7	0.87±0.02	0.06±0.01	4.28±2.3	0.15±0.03	0.72±0.1	5:1
	**OVA treated**	9.6±1.2	0.67±0.5	8.4±2.4	1.2±0.3	0.5±0.1	0.2±0.01	10:1
**α4-/-**	**Control**	9.92±2.3	0.8±0.1	0.07±0.001	9.85±3.4	0.03±0.001	0.8±0.1	12:1
	**OVA treated**	26.3±4.3	2.92±0.6	21.6±5.3	4.7±1.4	2.45±0.8	0.47±0.1	9:1
**β2-/-**	**Control**	21±2.5	11.2±1.5	0.53±0.1	20.5±2.4	0.45±0.02	10.75±4	1.8:1
	**OVA treated**	36±3.7	7.2±2.2	29.7±7	6.3±0.9	6.7±1.3	0.5±0.01	5:1

**Table 4C.** Blood

(x10^6^)		B220+	B220+IgM+ Mature plasma cells	B220+CD19+ Memory cells	B220+CD23+ Allergen specific plasma cells
**WT**	**Control**	2.8±0.6	1.84±0.5	0.27±0.12	0.79±0.2
	**OVA treated**	6.25±1	3.6±1.3	8.81±2.6	3.36±1.08
**α4-/-**	**Control**	5.32±1.7	5.6±1.9	1.23±0.7	2.13±0.75
	**OVA treated**	25.6±7.5	17.4±3.5	16.83±3.6	17.8±5.7
**β2-/-**	**Control**	3.7±2.3	15.25±6.7	0.56±0.01	0.2±0.1
	**OVA treated**	14.6±1.5	27.5±11.9	8.71±3.9	14.08±5.6

**Table 4D.** Blood

(x10^6^)		GR1^-^F4/80^+^	GR1^+^F4/80^+^	GR1^+^F4/80^+^			GR1^+^F4/80^-^
				Gr1^lo^F4/80^hi^	Gr1^lo^F4/80^lo^	Gr1^hi^F4/80^lo^	
**WT**	**Control**	0.6±0.5	2.6±0.9	0.66±0.01	0.31±0.01	0.014±0.001	0.72±0.13
	**OVA treated**	0.83±0.2	3.2±0.6	1.3±0.1	1.66±1.5	0.26±0.01	0.99±0.02
**α4-/-**	**Control**	0.5±2.1	6.23±1.4	3.3±0.2	1.36±0.25	0.024±0.001	0.78±0.03
	**OVA treated**	9.7±3.3	18.6±6.6	11.08+0. 5	6.7±12.9	0.314±0.01	10.7±1.97
**β2-/-**	**Control**	0.74±0.2	47.66±8.3	3.05±1.8	7.1±4.5	14.9±5	3.4±11
	**OVA treated**	5.2±0.8	54.1±3.04	8.23±0.9	19.9±3.74	25.86±2.6	19.81±3.97

**Table 5:** Total count (Lymphoid and myeloid cells) in BALf. Number of cells in PLO and SLO and tissue were calculated as tC and DC by hemocytometric analysis in a standard Neubauer’s Hemocytometer, DC was analyzed by double blind counting from H&E stained smears prepared in a cytospin (manufactured by Vision Scientific, South Korea, model Centurion Scientific C2 series) using Zeiss photo allotment and Axiostar plus software and by flow cytometry using BD flow cytometer (BD Accuri C6 cytometer) and analyzed by BD Accuri C6 software using monoclonal fluorochrome tagged antibody as mentioned in Materials & Methods. Table 5A. BALf

(x10^6^)		Total	T cells	B cells
**WT**	**Control**	0.87±0.01	0.009±0.0001	0.047±0.001
	**OVA treated**	8.5±0.4	1.05±0.001	1.398±0.15
**α4-/-**	**Control**	0.62±0.09	0.002±0.001	0.015±0.001
	**OVA treated**	0.82±0.01	0.009±0.001	0.04±0.001
**β2-/-**	**Control**	0.2±0.01	0.008±0.0001	0.013±0.0001
	**OVA treated**	0.72±0.03	0.06±0.0002	0.02±0.001

**Table 5B.** BALf

T cell subsets								
(x10^3^)		CD4+	CD8+	CD4+		CD8+		CD4:CD8
				memory	naive	memory	naive	
**WT**	**Control**	7.8±2.5	0.17±0.01	0	7.8±2.2	0	0.17±0.01	46:1
	**OVA treated**	1485±89	15±2.6	1448±54	36.5±7.1	14.5±2.7	0.46±0.01	98.6:1
**α4-/-**	**Control**	2±0.3	1±0.7	0.03±0.001	1.96±0.1	0.05±0.01	0.9±0.01	2:1
	**OVA treated**	7.7±1.2	0.26±0.1	2.09±0.1	5.6±0.1	0.006±0.001	0.25±0.05	29.6:1
**β2-/-**	**Control**	5±0.3	1±0.4	0.125±0.35	4.875±1.5	0.03±0.001	0.96±0.15	5:1
	**OVA treated**	6±0.2	2±0.8	1.69±0.8	4.3±1.7	0.8±0.002	1.16±0.5	3:1

**Table 5C.** BALf

(x10^3^)		B220+	B220+IgM+ Mature plasma cells	B220+CD19+ Memory cells	B220+CD23+ Allergen specific plasma cells
**WT**	**Control**	47±1	48.5±16	5.94±2.1	0.003±0.001
	**OVA treated**	1398±150	144.5±67	98.3±11.5	102.7±86
**α4-/-**	**Control**	15+ 1	16.2±6.3	13.6±6.4	0.01±0.001
	**OVA treated**	40±1	53.3±12	52±11	42±7
**β2-/-**	**Control**	13±1	8.62±1.5	4.6±1.13	0.002±0.001
	**OVA treated**	20±1	48.65±8.4	31.5±5.9	5.89±1.8

**Table 5D.** BALf

(x10^3^)		GR1^-^F4/80^+^	GR1^+^F4/80^+^	GR1^+^F4/80^+^			GR1^+^F4/80^-^
				Gr1^lo^F4/80^hi^	Gr1^lo^F4/80^lo^	Gr1^hi^F4/80^lo^	
**WT**	**Control**	13±3	350±120	55.7±11.6	52.2±31	33.05±11	305.7±15
	**OVA treated**	2100±98	977±98	215±98	302±156	760±2.5	796±210
**α4-/-**	**Control**	9.5±1.6	29.8±9.6	17.8±1.7	7.4±2.5	0.1±0.01	17.2±2.6
	**OVA treated**	17.8±1.08	426.5±120	260.26±34	81±13.6	6.8±1.2	34.2±11.9
**β2-/-**	**Control**	15±1	160±28	80±28	37±1.5	5±1.4	20±1.6
	**OVA treated**	120±31	560±23	250±7	70±13	90±3.7	25±1.8

**Table 5F.** BALf

(x10^3^)		Total	Lym	Mono	Mac	Mast	PMN	Eos
**WT**	**Control**	874±15	0	0	874±15	0	0	0
	**OVA treated**	8500±418	2817±145	1141±153	501±21	40±8	784±19	3880±45
**α4-/-**	**Control**	623±93	0	0	623±93	0	0	0
	**OVA treated**	827±17	0.6±0.01	26±0.4	2.6±1.1	0	36.3±9	0.9±0.01
**β2-/-**	**Control**	204±36	0	0	204±36	0	0	0
	**OVA treated**	718±15	75.9±12	141.8±0.5	64.9±0.6	0	66.06±14	0

**Table 6:** Total count (Lymphoid and myeloid cells) in LP. Number of cells in PLO and SLO and tissue were calculated as tC and DC by hemocytometric analysis in a standard Neubauer’s Hemocytometer, DC was analyzed by double blind counting from H&E stained smears prepared in a cytospin (manufactured by Vision Scientific, South Korea, model Centurion Scientific C2 series) using Zeiss photo allotment and Axiostar plus software and by flow cytometry using BD flow cytometer (BD Accuri C6 cytometer) and analyzed by BD Accuri C6 software using monoclonal fluorochrome tagged antibody as mentioned in Materials & Methods. Table 6A. LP

(x10^6^)		Total	T cells(CD3+)	B cells(B220+)
**WT**	**Control**	0.09±0.014	0.0135±0.001	0.004±0.001
	**OVA treated**	3.59±0.1	1.41±0.05	0.034±0.001
**α4-/-**	**Control**	0.07±0.03	0.0128±0.001	0.0038±0.0001
	**OVA treated**	1.322±0.04	0.209±0.01	0.014±0.00001
**β2-/-**	**Control**	0.44±0.12	0.0001±0.00001	0.006±0.00001
	**OVA treated**	5.35±0.97	0.004±0.0001	0.1±0.001

**Table 6B.** LP

T cell subsets								
(x10^3^)		CD4+	CD8+	CD4+		CD8+		CD4:CD8
				memory	naive	memory	naive	
**WT**	**Control**	13.3±0.85	0.2±0.01	0.95±0.02	12.4±0.07	0.01±0.01	0.2±0.01	66.5:1
	**OVA treated**	139±12.5	10±0.01	139±0.7	0.001	10±0.1	0.0001	14:1
**α4-/-**	**Control**	11.8±0.07	0.5±0.01	0.3±0.01	11.4±0.03	0.064±0.01	0.4±0.01	24:1
	**OVA treated**	202.8±15	6.2±4.8	6.7±3.5	196±7.1	0.08±0.03	6.2±1.5	33:1
**β2-/-**	**Control**	0.16±0.1	0.016±0.001	0.045±0.001	0.115±0.01	0.02±0.01	0.016±0.001	10:1
	**OVA treated**	2.9±3.3	0.9±0.01	1.8±0.02	1.1±0.005	0.8±0.01	0.1±0.004	3.2:1

**Table 6C.** LP

(x10^3^)		B220+	B220+IgM+ Mature plasma cells	B220+CD19+ Memory cells	B220+CD23+ Allergen specific plasma cells
**WT**	**Control**	4±1	8±5.5	6.7±2.2	0.002±0.0001
	**OVA treated**	34±1	28±1.78	29.5±1.8	31.2±15.5
**α4-/-**	**Control**	4±0.1	6±3.5	5.5±1.7	0.0001±0.0001
	**OVA treated**	14±0.001	18±8.5	15.7±3.6	6.3±3.45
**β2-/-**	**Control**	6±0.001	6±3.96	3.2±1.25	0.0001±0.0001
	**OVA treated**	100±1	94±12.4	76±13	2.5±1.2

**Table 6D.** LP

(x10^3^)		GR1^-^F4/80^+^	GR1^+^F4/80^+^	GR1^+^F4/80^+^			GR1+F4/80^-^
				Gr1^lo^F4/80^hi^	Gr1^lo^F4/80^lo^	Gr1^hi^F4/80^lo^	
**WT**	**Control**	1.67±0.5	23±12	0.28±0.1	6.9±5.6	1.07±0.08	6.45±0.35
	**OVA treated**	80±13.5	1430±5.7	83±0.4	430±12	50±24	680±78
**α4-/-**	**Control**	2.3±0.85	33.7±8.5	4.2±1.07	17±0.8	1.466±0.7	3.5±0.25
	**OVA treated**	20±0.2	328±15	24±0.05	60±0.45	3±0.35	118±0.5
**β2-/-**	**Control**	20±5.8	380±13.5	165±34	90±27	50±19	37±8
	**OVA treated**	97.5±32.5	4310±40	930±30	765±65	1480±190	920±80

**Table 7:** Total count (Lymphoid and myeloid cells) in trachea. Number of cells in PLO and SLO and tissue were calculated as tC and DC by hemocytometric analysis in a standard Neubauer’s Hemocytometer, DC was analyzed by double blind counting from H&E stained smears prepared in a cytospin (manufactured by Vision Scientific, South Korea, model Centurion Scientific C2 series) using Zeiss photo allotment and Axiostar plus software and by flow cytometry using BD flow cytometer (BD Accuri C6 cytometer) and analyzed by BD Accuri C6 software using monoclonal fluorochrome tagged antibody as mentioned in Materials & Methods. Table 7A. Trachea

(x10^6^)		Total	T cells(CD3+)	B cells(B220+)
**WT**	**Control**	18.1±9.8	2.8±0.3	2±0.7
	**OVA treated**	247.5±59.7	236.8±82.4	11.3±0.1
**α4-/-**	**Control**	54±2.37	1.9±0.2	10.6±0.8
	**OVA treated**	189.8±5.8	69.2±1	16.9±0.3
**β2-/-**	**Control**	283±18.9	8.65±0.4	5.7±0.7
	**OVA treated**	474±3.5	34.5±2.35	47.4±3.4

**Table 7B.** Trachea

T cell subsets								
(x10^3^)		CD4+	CD8+	CD4+		CD8+		CD4:CD8
				memory	naive	memory	naive	
**WT**	**Control**	2.1±0.3	0.7±0.05	0.03±0.0001	2.07±0.1	0.002±0.001	0.5±0.01	3:1
	**OVA treated**	177.5±0.8	60±0.1	169.8±1.14	0.7±0.03	57.36±1.1	2.5±0.8	3:1
**α4-/-**	**Control**	1.5±0.03	0.4±0.01	0.001±0.0001	1.46±0.1	0.001±0.001	0.4±0.01	3.75:1
	**OVA treated**	57.7±0.16	11.5±0.02	47.3±1.6	10.4±0.5	5.1±1.6	4.8±0.4	5:1
**β2-/-**	**Control**	6.2±0.01	0.2±0.04	0.003±0.0001	6±0.05	0.001±0.001	0.2±0.01	31:1
	**OVA treated**	27±0.4	7.5±1.3	21.3±7.5	5.7±0.12	3.6±2.6	3.7±0.5	3.6:1

**Table 7C.** Trachea

(x10^3^)		B220+	B220+IgM+ Mature plasma cells	B220+CD19+ Memory cells	B220+CD23+ Allergen specific plasma cells
**WT**	**Control**	2±0.7	0.04±0.001	0.03±0.001	0.01±0.001
	**OVA treated**	11.3±0.1	0.8±0.01	1.5±0.6	3.4±0.5
**α4-/-**	**Control**	10.6±0.8	0.01±0.001	0.06±0.001	0.02±0.07
	**OVA treated**	16.9±0.3	0.6±0.5	3.5±1.5	4.7±0.8
**β2-/-**	**Control**	5.7±0.7	0.1±0.04	0.04±0.001	0.08±0.001
	**OVA treated**	47.4±3.4	0.75±0.01	4.6±1.7	5.2±0.65

**Table 7D.** Trachea

(x10^3^)		GR1-F4/80+	GR1+F4/80+	GR1+F4/80-
**WT**	**Control**	0.2±0.02	1.6±0.5	12±2.5
	**OVA treated**	18±1.7	34.5±0.5	9.8±2.4
**α4-/-**	**Control**	0.03±0.001	41.5±13	15±0.75
	**OVA treated**	7±0.5	56±12.5	162.6±8.9
**β2-/-**	**Control**	0.05±0.001	135.5±20	27±0.5
	**OVA treated**	9±2.8	335±18.5	123±7.5

**Table 8:** Total count (Lymphoid and myeloid cells) in spleen. Number of cells in PLO and SLO and tissue were calculated as tC and DC by hemocytometric analysis in a standard Neubauer’s Hemocytometer, DC was analyzed by double blind counting from H&E stained smears prepared in a cytospin using Zeiss photo allotment and Axiostar plus software and by flow cytometry using BD flow cytometer and analyzed by BD Accuri C6 software using monoclonal fluorochrome tagged antibody as mentioned in Materials & Methods. Table 8A

(x10^6^)		Total	T cells(CD3+)	B cells(B220+)
**WT**	**Control**	83.95±25	38.87±21	43.08±2
	**OVA treated**	113±21.83	58.56±13.2	67.02±6.03
**α4-/-**	**Control**	123.81±18.97	47.73±7.6	63.86±12.6
	**OVA treated**	358.8±8.5	59.31±15	76.96±12
**β2-/-**	**Control**	142.5±42.98	52.94±5.87	71.03±3
	**OVA treated**	290.84±54	69.22±15.2	87.54±19.24

**Table 8B.** Spleen

T cell subsets								
(x10^6^)		CD4+	CD8+	CD4+		CD8+		CD4:CD8
				memory	naive	memory	naive	
**WT**	**Control**	25.33±0.323	10.53±2.01	1.05±0.37	20.13±1.19	0.2±0.07	10.33±4.5	2:1
	**OVA treated**	52.82±9.05	8.79±2.2	39.63±7.79	12.19±2.53	6.56±0.78	2.23±0.76	3.7:1
**α4-/-**	**Control**	34.25±6.56	5.18±1.02	3.76±5.04	30.49±5.89	0.41±0.01	4.77±2.1	6.6:1
	**OVA treated**	30.59±10.09	14.46±4.3	26.3±8.43	4.23±1.19	12.31±9.5	2.15±1.08	2:1
**β2-/-**	**Control**	34.8±0.06	6.93±0.04	0.696±0.43	34±7.8	0.22±0.17	6.71±3.9	5:1
	**OVA treated**	56.39±0.67	11.9±2.67	28.27±2.13	25.12±4.8	8.05±2.2	5.22±1.3	4.7:1

**Table 8C.** Spleen

(x10^6^)		B220+	B220+IgM+ Mature plasma cells	B220+CD19+ Memory cells	B220+CD23+ Allergen specific plasma cells
**WT**	**Control**	43.08±2	25.3±1.8	1.018±0.56	0.56±0.12
	**OVA treated**	67.02±6.03	39.4±4.8	54.69±32.16	21.25±8.1
**α4-/-**	**Control**	63.86±12.6	42.8±15.3	15.4±3.6	1.71±0.85
	**OVA treated**	76.96±12	161.3±67.8	51.87±6.95	28.76±4.6
**β2-/-**	**Control**	71.03±3	65.5±36.7	2.2±1.5	1.65±0.53
	**OVA treated**	87.54±19.24	104±65.3	70.7±11.7	57.69±31.27

## Discussion

**Figures** **1**&**2** show the diagrammatic representation of the basic study protocols of transplantation for hematopoietic reconstitution and development of preclinical asthma respectively. C57Bl/6J mouse from NIN under permission from IAEC (University of Washington, Seattle, USA) and Departmental Animals Ethics Committee (Dept. of Zoology, University of Calcutta, India). Using this basic template of the study protocol of acute allergic asthma, migration of cells under no-disease condition (“clean” Rag2γc-/- where progenitors were killed off by lethal γ-irradiation and bone marrow cells from donors were allowed to repopulate all PLO and SLO) and under diseased condition where two genotype knockout mice were used and acute allergic asthma induced to track and study the various nuances of cell migration in the different PLO and SLO and their subsequent recruitment to the pulmonary tissue for orchestrating inflammation.

At the outset, we must consider the significance of the donors that were chosen and the recipients’ pretreatment before transplantation. It may be conclusively deduced from **Fig**. **3** that some SLO are resistant to ablation of this integrin. The reason may be important metabolomic and transcriptomic pathways that disallows this resulting in the immunosecretome being readjusted and repositioned such that ablation of α4 is compensated. **Fig**. **4** however, unambiguously excludes BMC from such non-permissiveness to ablation ensuring a 100% αa free reconstitution. As seen in previous publications, (Banerjee et al., 2009; Banerjee et al., 2008; Ray Banerjee, 2007; Scott et al., 2003), α4 has regulatory roles in mobilization, homing, and engraftment which explains their higher progenitor number in PLO (BM), circulation (PB), and spleen (SLO) in the KO groups but not appreciable numbers post OVA as was found in the WT (**Fig**. **5**).

This is key. In all other secondary lymphoid organs assessed, namely, MLN, LNI and LNX, except CLN, pattern of cellular traffic in WT vs. KO groups and control vs. OVA treated groups do not follow a uniform pattern or any “trend”. The complete oppositeness of progenitor number modulation post-OVA in the two genotype groups indicate their diverse pathways action as enumerated in (Banerjee et al., 2009; Ray Banerjee, 2007; Ulyanova et al., 2007). But what is interesting here is the complete separation of the cell traffic (direction and number) as evident from **Tables** **1**-**8** and **Fig**. **6A**-**H**. The picture of Tand B-cell distribution in spleen is more in line with what is present in PB, and contrasts that of BM described above most likely because of longer retention and maturation of these cells in the splenic environment compared to BM. CD40+ (dendritic cells) were lower in all organs except the spleen, where the proportion, but not the total number, was low.

**Fig**. **7** shows the pathways of induction of differentiation of various mature functionally active immune cells that are key to inflammation. Additionally, the figure also outlines the switch from progenitor (pluripotent cells) towards lineage commitment and final differentiation into competent immune cells responsible for the immune activities within tissues. Important to note are the cytokines and other factors that not only induce differentiation but also attracts the cells from their sites of synthesis (PLO) to their sites of maturation (SLO) down to the tissues where they either perish in the onslaught or return to PLO or SLO (homing) with valuable information to be encoded as “central memory” cells that either become the effectors as and when they are recalled (mobilization) in the future during another exigency. **Fig**. **8** represents ramifications of the network of growth factors and their receptors, adhesion and signalling molecules and transcription factors, maturation, morphogenic and their guidance molecules, ECM proteins and proteinases. **Fig**. **9** shows schematically, the various functional cells populating the PLO, SLO and migrating to tissues, the known immune system lymphoid and myeloid subpopulations and their inducing cytokines and growth factors. **Fig**. **10** depicts the major PLO, SLO in humans and that denotes their exact counterparts in mouse as they will be extrapolated to represent.

Mortality being low and repopulation of transplanted cells being 100% by 48 weeks, we can say safely that the transplanted BMC did their job well, that is they found the regions to home to and then they settled down there and all progenitors generated new clones and differentiated into the type of cells that the tissues needed. In other words there was induction of differentiation *in situ*.

As for the rate limiting steps which are key in cellular traffic resulting in onset and development of the actual disease, α4 was found to be a critical regulatory factor for myeloid cellular traffic compared to lymphoid. (**Table** **1**) Overall, both integrins seem to have regulatory roles in lymphoid and nonlymphoid cell migration to blood. The number of cells in BALf is the most striking and as described in (Banerjee et al., 2009; Banerjee et al., 2008; Ray Banerjee, 2011b; Ulyanova et al., 2007) that while α4 principally controls sensitization and signaling, β2 mainly control the migration from lung parenchyma to interstitium without which onset of the asthma immunopathology cannot occur. **Table** **2** reveals a curious thing. A subpopulation of cells GR1+F4/80+ (GR1^hi^ F4/80^hi^), probably newly formed macrophages migrated to pulmonary tissue from BM, which is a minus percent recruited from blood in normal untreated mice, shoot up in both KO groups, notwithstanding their lack of recruitment post-OVA.

**Fig 6A.**
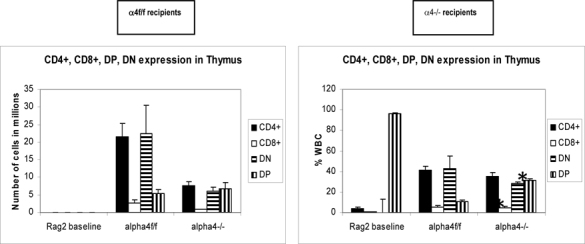
Cellularity in thymus

**Figure 6B.**
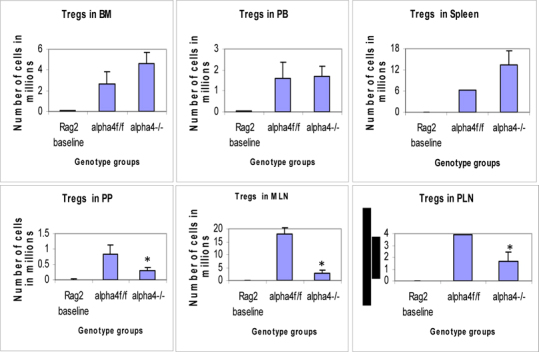
Distribution of different development stages of B and T cells in BM, PB and Spleen. Graph of cell markers in BM, PB and Spleen, CD3+

**Figure 6C.**
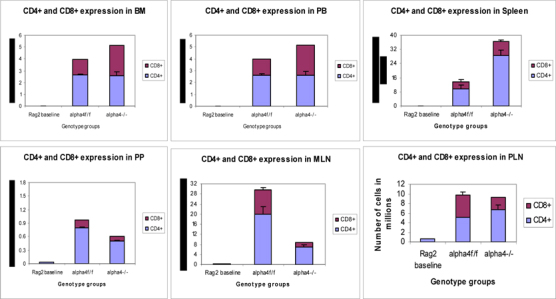
Distribution of different development stages of T cell subsets in BM, PB and Spleen; Graph of cell markers in BM, PB and Spleen, CD4+, CD8+

**Figure 6D.**
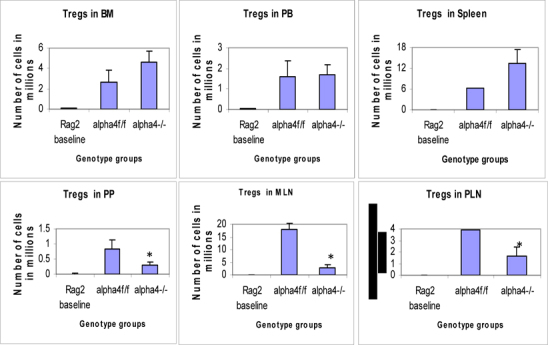
Distribution of different developmental stages of T cells in BM, PB and spleen. Graph of cell markers in BM, PB and Spleen, CD4+CD25+ Treg.

**Figure 6E.**
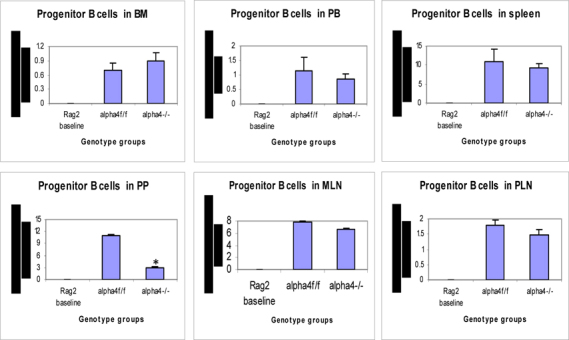
Distribution of different developmental stages of T cells in BM, PB and spleen. Graph of cell markers in BM, PB and Spleen, Progenitor B cells

**Figure 6F.**
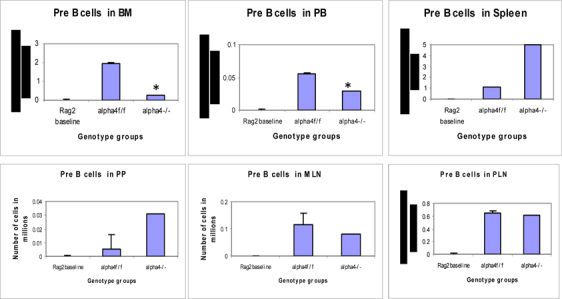
Distribution of different developmental stages of T cells in BM, PB and spleen. Graph of cell markers in BM, PB and Spleen, Pre B cells B220+CD34-

**Figure 6G.**
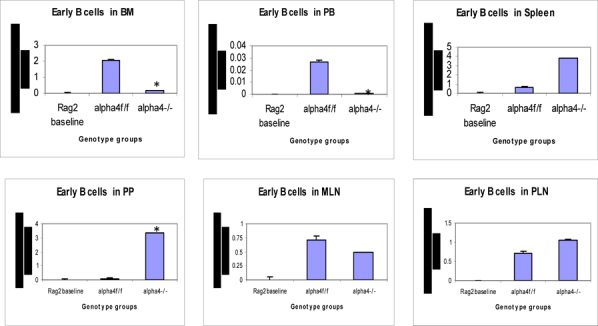
Distribution of different developmental stages of T cells in BM, PB and spleen. Graph of cell markers in BM, PB and Spleen, Pro B cells – Early B cells

**Figure 6H.**
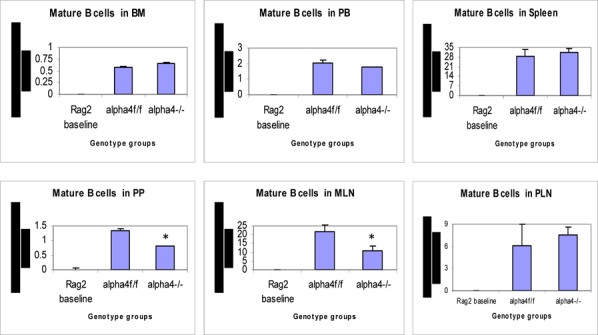
Distribution of different developmental stages of T cells in BM, PB and spleen. Graph of cell markers in BM, PB and Spleen, Mature B cells (B220+IgM+)

**Figure 7.**
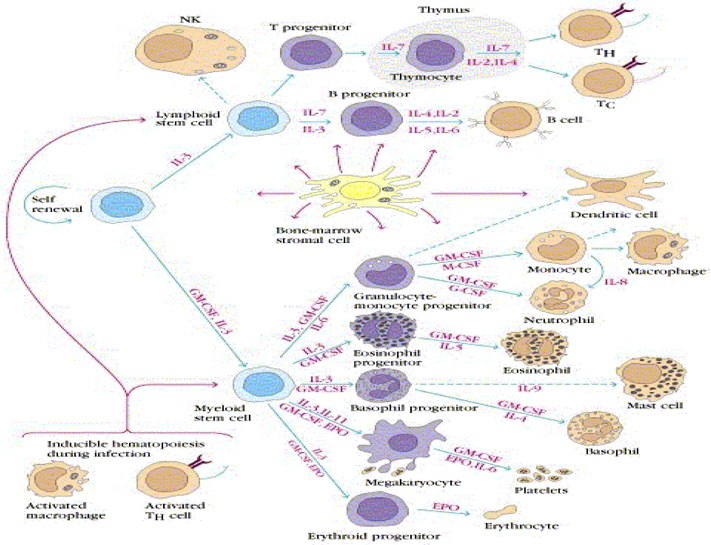
Mediators of differentiation pathways from stem cells.

**Figure 8.**
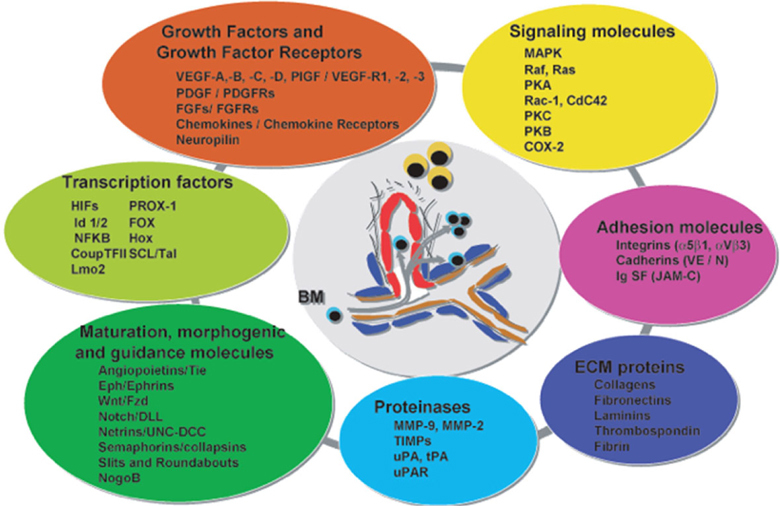
Ramifications of myriad factors operative in inflammation in complex network.

**Figure 9.**
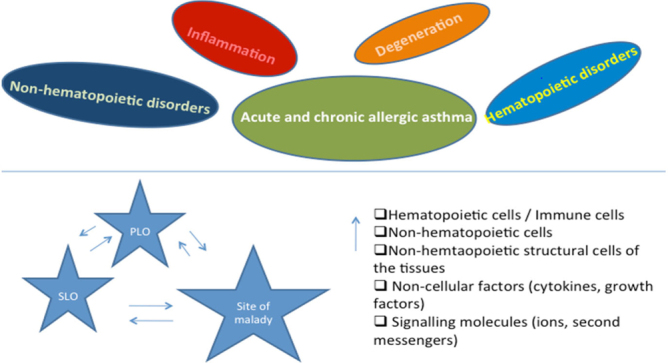
Schematic representation of the key inflammasomes and their pathways.

**Figure 10.**
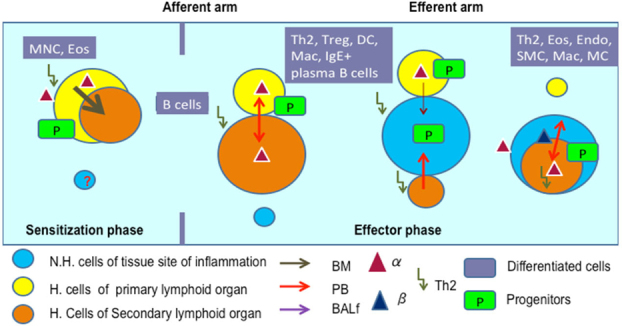
Summary findings and conclusion - Tentative scheme of cellular traffic.

This may indicate either a sub-clinical inflammation in the KO mice which is spontaneous in nature or it may be cells newly recruited from bone marrow with other more extraneous and somewhat fortuitous functions. This group need better characterization. The GR1+F4/80- myeloid population which is definitely the new recruits from BM which have still not lost their GR1 expression may be short-lived granulocytes which show similar trend as this preceding group. The significance may be better grasped in the following tables where trends in PLO top SLO migration become clearer. The rate limiting cells are therefore lymphoid cells as they migrate from blood to lung tissue and thence to the interstitial spaces where the actual gas exchange occurs and this step is almost exclusively controlled by the β2 integrin. α4 controls the lymphoid signaling while β2 controls myeloid migration. So these are the rate limiting cells from PLO to tissue. **Table** **3** reveals conclusively that although in α4-/- BM, synthesis and sequestration of T cells fall quite tremendously and post-OVA their number is amplified manifolds more than in WT. As mentioned in (Scott et al., 2003), this may either be due to a lack of “settling down” on the stroma of the PLO or a genuine increase in synthesis of these cells but it certainly means that there is possibly no impairment in the upstream IL-4-IL-4R and IL-5-induced recruitment from bone marrow in its absence. Data shown in **Table** **7D** coupled with data presented in **Fig**. **6A** highlight that α4 is probably key to the Th1-Th2 balance.

In **Table** **5**-**7C**, data shows that although IL-4/5 induced cell recruitment is probably still operative despite absence of β2- cells from these B cells, they are unable to cross the number threshold and therefore insufficient to mount a viable pathophysiological circuit. (Banerjee et al., 2008; Farrell, 2003; Lee et al., 2003; Ray Banerjee, 2007; Scott et al., 2003; Ulyanova et al., 2007; Weber et al., 1992). There may be a role for epithelial progenitors, circulating and niche-dwelling (Gomperts et al., 2006) which is beyond the scope of this paper and is being currently investigated.

## Summary findings & conclusion- Highlights

### Observations

1. α-/- cells are slower to reconstitute
2. Increased hematopoiesis in all primary & secondary lymphoid organs in KO gr
3. Myeloid repopulation was similar in both groups
4. B cells, pro B cells, pre B cells, mature B cells significantly decreased in KO gr
5. Total T cells increased but activated T cells decreased in KO gr
6. Repopulation of thymus was significantly impaired in KO gr
7. CD+CD8+ was more in thymus and CD+ differentiation was skewed in KO gr
8. OVA-induced acute allergic asthma phenotype was impaired in all aspects
9. All PLNs show decreased mature B cells
10. Enhanced tendency of memory T cells (CD45RC-CD4+) to migrate to LNs in KO gr
11. Enhanced tendency of memory T cells (CD45RC+CD4+) to migrate to spleen & thymus
12. Functionality & homing impairment in KO gr
13. α-/- probably do not have a role to play during response to OVA *per se*, and neither to specifically T cell proliferation in response to the upstream cytokine signaling in response to OVA.

### Salient findings and clinical implications

Various small molecules inhibitors for α4 integrin such as Tysabri (Roche) and integrin Beta2/CD18 antibodies products manufactured by Biolegend and Novus Biologicals and others which are specifically designed for modulation of the target molecule must take into account targeting strategies along with the timing of drug activation in a particular tissue (in pro drug formulation, through nano-vehicles for timed and tissue specific delivery. Positioning of PLO, SLO and inflamed tissue as nodes of cellular and information traffic and the network operative in specific temporal templates is crucial information for the success of such drugs. The role of α4 and β2 are very much cell specific and tissue specific, for example their role in bone marrow, blood, lung, interstitial tissue spaces, trachea, and lymph nodes are not uniform. The “kicking in” is initiated possibly by α4 during initiation and “priming” of the B cells and T cells.

1. The “changing gear” is possibly orchestrated by β2 during the effector phase of the disease development.

It is through the detailed mapping of such “switching on – switching off” information throughput of the cellular and non-cellular role players, translational and personalized medicine may devise effective strategies in disease amelioration.

## Conclusion

α4 &β2 integrins control cellular migration of all lymphoid subpopulations from BM (site of poiesis) to PLO and of some sub-populations from PLO to SLO but has no effect on myelopoiesis or homing and functionality.

## Abbreviations

OVA: ovalbumin
BM: bone marrow
PB: peripheral blood
BALf: bronchoalveolar lavage fluid
LP: lung parenchyma
LNX: Axillary lymph node
LNI: Inguinal lymph node
MLN: mesenteric lymph node
PP: Peyer’s patch
AHR: airway hyper-reactivity/responsiveness
i.t.: intra-tracheal
i.v.: intravenous
i.p.: intraperitoneal
H&E: Hematoxylin and Eosin
Penh: enhanced pause
WBP: whole body plethysmography
KO: knockout

## References

[CR1] Banerjee E. (2014). Looking for the elusive lung stem cell niche. Transl Respir Med.

[CR2] Banerjee E., Henderson W. (2012). Characterization of lung stem cell niches in a mouse model of bleomycin-induced fibrosis. Stem Cell Research & Therapy.

[CR3] Banerjee E., Henderson W.R. (2012). Defining the molecular role of gp91phox in the immune manifestation of acute allergic asthma using a preclinical murine model. Clinical and Molecular Allergy.

[CR4] Banerjee E.R., Henderson W.R. (2013). Role of T cells in a gp91phox knockout murine model of acute allergic asthma. Allergy, Asthma & Clinical Immunology.

[CR5] Banerjee E.R., Jiang Y., Henderson W.R., Latchman Y., Papayannopoulou T. (2009). Absence of α4 but not β2 integrins restrains development of chronic allergic asthma using mouse genetic models. Experimental Hematology.

[CR6] Banerjee E.R., Laflamme M.A., Papayannopoulou T., Kahn M., Murry C.E., Henderson W.R. (2012). Human Embryonic Stem Cells Differentiated to Lung Lineage-Specific Cells Ameliorate Pulmonary Fibrosis in a Xenograft Transplant Mouse Model. PLoS One.

[CR7] Banerjee E.R., Latchman Y.E., Jiang Y., Priestley G.V., Papayannopoulou T. (2008). Distinct changes in adult lymphopoiesis in Rag2–/– mice fully reconstituted by α4- deficient adult bone marrow cells. Experimental Hematology.

[CR8] Broide D.H., Sullivan S., Gifford T., Sriramarao P. (1998). Inhibition of Pulmonary Eosinophilia in P-Selectin- and ICAM-1- deficient Mice. American Journal of Respiratory Cell and Molecular Biology.

[CR9] Calverley P.M.A., Anderson J.A., Celli B., Ferguson G.T., Jenkins C., Jones P.W., Yates J.C., Vestbo J. (2007). Salmeterol and Fluticasone Propionate and Survival in Chronic Obstructive Pulmonary Disease. New England Journal of Medicine.

[CR10] Chin J., Hatfield CA, Winterrowd GE, Brashler JR, Vonderfecht SL, Fidler SF, Griffin RL, Kolbasa KP, Krzesicki RF, Sly LM, Staite ND, Richards IM (1997). Airway recruitment of leukocytes in mice is dependent on alphα4-integrins and vascular cell adhesion molecule-1. Am J Physiol.

[CR11] Cushley M.J., Tattersfield A.E., Holgate S.T. (1983). Inhaled adenosine and guanosine on airway resistance in normal and asthmatic subjects. British Journal of Clinical Pharmacology.

[CR12] Czarnobilska E, O. (2005). Eosinophil in allergic and non-allergic inflammation. Przegl Lek.

[CR38] Davidson E., Liu J.J., Sheikh A. (2010). The impact of ethnicity on asthma care. Primary Care Respiratory Journal.

[CR13] Erlandsen S.L., Hasslen S.R., Nelson R.D. (1993). Detection and spatial distribution of the beta 2 integrin (Mac-1) and Lselectin (LECAM-1) adherence receptors on human neutrophils by high-resolution field emission SEM. Journal of Histochemistry & Cytochemistry.

[CR14] Farrell R. (2003). Glucocorticoid resistance in inflammatory bowel disease. Journal of Endocrinology.

[CR15] Gomperts B.N., Belperio J.A., Rao P.N., Randell S.H., Fishbein M.C., Burdick M.D., Strieter R.M. (2006). Circulating Progenitor Epithelial Cells Traffic via CXCR4/CXCL12 in Response to Airway Injury. The Journal of Immunology.

[CR16] Henderson W.R., Chi E.Y., Albert R.K., Chu S.J., Lamm W.J., Rochon Y., Jonas M., Christie P.E., Harlan J.M. (1997). Blockade of CD49d (alpha4 integrin) on intrapulmonary but not circulating leukocytes inhibits airway inflammation and hyperresponsiveness in a mouse model of asthma. Journal of Clinical Investigation.

[CR17] Henderson W.R., Tang L.-O., Chu S.-J., Tsao S.-M., Chiang G.K.S., Jones F., Jonas M., Pae C., Wang H., Chi E.Y. (2002). A Role for Cysteinyl Leukotrienes in Airway Remodeling in a Mouse Asthma Model. Am J Respir Crit Care Med.

[CR18] Holgate S.T. (2007). The epithelium takes centre stage in asthma and atopic dermatitis. Trends in Immunology.

[CR19] Laberge S., Rabb H., Issekutz T.B., Martin J.G. (1995). Role of VLA-4 and LFA-1 in Allergen-Induced Airway Hyperresponsiveness and Lung Inflammation in the Rat. Am J Respir Crit Care Med.

[CR20] Lee S.-H., Prince J.E., Rais M., Kheradmand F., Shardonofsky F., Lu H., Beaudet A.L., Smith C.W., Soong L., Corry D.B. (2003). Differential requirement for CD18 in T-helper effector homing. Nature Medicine.

[CR21] Murphy D.M. (2010). Recent Advances in the Pathophysiology of Asthma. CHEST Journal.

[CR22] Nakajima H. (1994). Role of vascular cell adhesion molecule 1/very late activation antigen 4 and intercellular adhesion molecule 1/lymphocyte function-associated antigen 1 interactions in antigen-induced eosinophil and T cell recruitment into the tissue. Journal of Experimental Medicine.

[CR23] Poole P. (2001). Oral mucolytic drugs for exacerbations of chronic obstructive pulmonary disease: systematic review. BMJ.

[CR24] Ray Banerjee E. (2011). Triple selectin knockout (ELP-/-) mice fail to develop OVA-induced acute asthma phenotype. Journal of Inflammation.

[CR25] Ray Banerjee, Jr W.R. (2011). NADPH oxidase has a regulatory role in acute allergic asthma. Journal of Advanced Laboratory Research in Biology.

[CR26] Ray Banerjee E.J., Henderson Y., Scott L.M, Papayannopoulou T (2007). Alpha4 and beta2 integrins have non-overlapping roles in asthma development, but for optimal allergen sensitization only alpha4 is critical. Exp Hematol.

[CR27] Rennard S.I., Fogarty C., Kelsen S., Long W., Ramsdell J., Allison J., Mahler D., Saadeh C., Siler T., Snell P (2007). The Safety and Efficacy of Infliximab in Moderate to Severe Chronic Obstructive Pulmonary Disease. Am J Respir Crit Care Med.

[CR28] Schneider T., Issekutz T.B., Issekutz A.C. (1999). The Role of α 4 (CD49d) and β 2 (CD18) Integrins in Eosinophil and Neutrophil Migration to Allergic Lung Inflammation in the Brown Norway Rat. American Journal of Respiratory Cell and Molecular Biology.

[CR29] Scott L.M., Priestley G.V., Papayannopoulou T. (2003). Deletion of 4 Integrins from Adult Hematopoietic Cells Reveals Roles in Homeostasis, Regeneration, and Homing. Molecular and Cellular Biology.

[CR30] Sharma P H.A. (2009). Emerging molecular targets for the treatment of asthma. ndian J Biochem Biophys.

[CR31] Takizawa H. (2007). Novel Strategies for the Treatment of Asthma. Recent Patents on Inflammation & Allergy Drug Discovery.

[CR32] Troosters T., Celli B., Lystig T., Kesten S., Mehra S., Tashkin D.P., Decramer M. (2010). Tiotropium as a first maintenance drug in COPD: secondary analysis of the UPLIFT(R) trial. European Respiratory Journal.

[CR33] Ulyanova T., Priestley G., Banerjee E., Papayannopoulou T. (2007). Unique and redundant roles of α4 and β2 integrins in kinetics of recruitment of lymphoid vs myeloid cell subsets to the inflamed peritoneum revealed by studies of genetically deficient mice. Experimental Hematology.

[CR34] Weber P., Koch M., Heizmann W.R., Scheurlen M., Jenss H., Hartmann F. (1992). Microbic superinfection in relapse of inflammatory bowel disease. Journal of clinical gastroenterology.

[CR35] Woodside D.G., Vanderslice P. (2008). Cell Adhesion Antagonists. BioDrugs.

[CR36] Yang G. (2006). Validation of verbal autopsy procedures for adult deaths in China. International Journal of Epidemiology.

[CR37] ZF U. (2007). Journal of The Association of Physicians of India.

